# Selective lymphadenectomy in endometrial cancer: Retrospective analysis of morbidity and survival data at a tertiary care centre

**DOI:** 10.12669/pjms.314.7593

**Published:** 2015

**Authors:** Uzma Chishti, Aliya B. Aziz, Munazza Akhtar, Sana Sheikh

**Affiliations:** 1 Dr. Uzma Chishti, Senior Instructor, Department of Obstetrics and Gynaecology, Aga Khan University Hospital, Karachi, Pakistan; 2 Dr. Aliya B. Aziz, Assistant Professor, Department of Obstetrics and Gynaecology, Aga Khan University Hospital, Karachi, Pakistan; 3 Dr. Munazza Akhtar, Senior Instructor, Department of Obstetrics and Gynaecology, Aga Khan University Hospital, Karachi, Pakistan

**Keywords:** Endometrial cancer, Lymphadenectomy

## Abstract

**Objective:**

To compare perioperative morbidity and survival data between patients with early-stage endometrial cancer who did or did not undergo selective lymphadenectomy.

**Methods:**

Retrospective analysis of 180 patients with early-stage endometrial carcinoma treated between 1999 and 2008 was performed in Aga Khan University Hospital, Karachi, Pakistan.

**Results:**

Data from 180 patients were analysed. The selective lymphadenectomy group contained 108 women (60%) and the no lymphadenectomy group contained 72 women (40%). The median number of lymph nodes removed was 9. The mean age and extent of disease, as assessed by staging, tumour size, myometrial invasion, and lymphovascular invasion were comparable between groups. Upstaging of the disease to stage 3 and 4 occurred in 11% of patients in the lymphadenectomy group. There were no significant differences in the medical or surgical complications between groups. At a median follow-up of 26 months, both groups had comparable survival (lymphadenectomy versus no lymphadenectomy: 34 versus 32 months). Similar survival was noted for patients who underwent the removal of more or less than 5 pelvic lymph nodes.

**Conclusion:**

Selective lymphadenectomy offers the advantage of improved surgical staging but no therapeutic benefit in terms of overall survival.

## INTRODUCTION

Worldwide, endometrial carcinoma is the second most common female genital tract cancer, after cervical cancer. In Asia, it is the most common female genital tract malignancy, accounting for nearly 41% of all new gynaecological cancers. In Pakistan, endometrial cancer is the third most common genital tract malignancy, after cancer of the cervix or ovary. Its incidence is rising because of the increased life expectancy and prevalence of obesity observed in the recent years.

Most cases (95%) of uterine carcinoma occur in women over 40 years of age, usually in the sixth and seventh decades of life. The overall lifetime risk of developing endometrial carcinoma is 2.5%.^1^ Seventy-five percent of endometrial carcinomas is confined to the uterus (clinical stage 1) at the time of diagnosis. Endometrial carcinoma generally carries an excellent prognosis in terms of curability and recurrence. There is an increasing emphasis on surgico-pathologic staging, which includes the need for pelvic and para-aortic lymphadenectomy to accurately identify lymphatic spread. However, it is not well established how this information alters prognosis and whether it can guide the use of adjuvant therapies. The major concerns regarding lymphadenectomy, therefore, include whether it is necessary, the extent of lymph node dissection required, and whether it can add any therapeutic benefit.^2^-^5^

The majority of women with endometrial carcinoma are considered low-risk for nodal disease at presentation, in which low risk is defined as disease limited to the corpus uteri, a histologic grade of 1 or 2, endometrioid histology, and myometrial invasion of < 50%.^6^ However, justification for performing lymph node dissection is two-fold. One reason is to eradicate lymph node disease, if present, and the second is to exclude patients with negative nodes on histopathology from adjuvant therapy, thus sparing them from the adverse effects of radiotherapy.

The present study was conducted to compare morbidity and survival data in patients with early-stage endometrial cancer who did or did not undergo selective lymphadenectomy. Our results may be helpful in developing institutional and national guidelines for the standard management of these patients.

## METHODS

After the approval of our institutional review board, we conducted a retrospective analysis of all patients who underwent surgical treatment at Aga Khan University Hospital, Karachi, Pakistan, for suspected early-stage endometrial cancer from January 1999 to December 2008. Patients who received neoadjuvant chemotherapy or were noted to have extra-uterine disease at the time of laparotomy were excluded from the study. The decision to perform lymphadenectomy or not depended on the clinical and radiological assessment of the extent of disease, medical comorbidities, and New York Heart Association class of the patient. This decision was primarily based on the individual surgeon’s assessment and preference.

Selective pelvic lymphadenectomy involved removing lymphatic tissue from the anterior and medial surfaces of the iliac vessels and from the obturator fossa superior to the obturator nerve. The decision to remove para-aortic lymph nodes was based on radiological assessment, as well as the grade of the disease. This procedure involved removing the precaval and right and left aortic lymphatic tissue to the level of the inferior mesenteric artery.

Medical records for all patients were reviewed, and relevant demographic, clinical, surgical, pathologic, and follow-up information was acquired. Overall survival was compared between the two treatment groups (i.e., those who underwent selective lymphadenectomy versus those who did not undergo this procedure). Perioperative morbidity parameters (e.g., duration of surgery, estimated blood loss, and organ injury) and postoperative complications (e.g., infections, thromboembolism, lymphocysts, and lymphoedema) were also compared between the two groups.

SPSS version 19 was used for data recording and analysis. Associations between categorical covariates were assessed using chi-square tests, whereas the t-test was used to assess group differences for continuous variables. A two-sided p-value of ≤ 0.05 was considered statistically significant. Survival curves were estimated using the Kaplan–Meier method, and the log-rank test was used to compare curves between the groups.

## RESULTS

A total of 180 patients treated for early-stage endometrial carcinoma at our institution from 1999 to 2008 met our eligibility criteria. Of these, 108 patients (60%) underwent selective lymphadenectomy (Group A) and the remaining 72 patients (40%) did not undergo selective lymphadenectomy (Group B). In Group A, the median number of lymph nodes removed was 9 and the median number of para-aortic nodes removed was 2.

The clinico-pathological characteristics of patients in both groups are shown in Table-I. These characteristics were comparable between groups, except for the proportion of patients undergoing lymphadenectomy in the different pathologic grade groups (p-value < 0.01). Overall, 45% of our patients had grade 1 disease and 42% of these patients underwent lymphadenectomy. By contrast, 34% of our patients had grade 2 disease and 79% of these patients underwent lymphadenectomy; 10% of our patients had grade 3 disease and 83% of these patients underwent lymphadenectomy. The extent of the disease, as assessed by stage (p = 0.06), tumour size (p = 0.36), myometrial invasion (p = 0.27), and lymphovascular invasion (p > 0.99), was comparable between the groups. (Table-I)

**Table-I:** Demographic and Clinical Characteristics of the Study Groups.

	Total(N=180)	Lymphadenectomy (Group A)(n=108)	No Lymphadenectomy (Group B)(n=72)	P-value
Age (years)	57.2 ±10.7	57.6 ± 10.97	56.5±10.39	0.40
*Parity*				
Nullipara	36 (20)	18 (50)	18 (50)	0.187
Any parity	144 (80)	90 (62.5)	54 (37.5)	
*Body mass index*				
Non-obese (<25)	32 (18.6)	18 (56)	14 (44)	0.80
Obese (>25)	140 (81.4)	83 (59)	57 (41)	
FIGO stage *				
Stage I	151 (83.9)	87 (57)	64 (42.4)	0.06
Stage II	10 (5.6)	9 (90.0)	1 (10.0)	
Stage III	15 (8.3)	11 (73.3)	4 (27)	
Stage IV	4 (2.2)	1 (25)	3 (75)	
*FIGO grade*				
Grade 1	82 (45.6)	34 (41.5)	48 (58.5)	< 0.01
Grade 2	61 (33.9)	48 (78.7)	13 (21.3)	
Grade 3	18 (10)	15 (83.3)	3 (16.7)	
*Tumour size (cm)*				
< 2	46 (25.7)	24 (52.2)	22 (47.8)	0.36
> 2	132 (73.7)	83 (62.9)	49 (37)	
*Myometrial invasion*				
None	18 (10)	11 (61)	7 (38.9)	0.27
Less than half	107 (59.4)	60 (56)	47 (44)	
More than half	55 (30.6)	37 (67)	18 (33)	
*Lymphovascular invasion*				
No	168 (93.3)	102 (60.7)	66 (39.3)	> 0.99
Yes	11 (6.1)	5 (45.5)	6 (54.5)	
*Number of lymph nodes removed*				
< 5	31 (29)			
> 5	77 (71)			

Data are presented as number (percentage) or mean ± standard deviation.

Abbreviations: FIGO, International Federation of Gynecology and Obstetrics.

Postoperative complications were noted in 21 patients (11.6%). (Table-II) Of these, 15 patients (14%) were in Group A and 6 patients (8%) were in Group B. There were no significant differences in medical or surgical complications between the groups. The most common complication was wound infection, which was observed in 10 patients. The median duration of surgery (160 minutes in group A versus 141 minutes in group B) and mean estimated blood loss (474 mL in group A versus 606 mL in group B) were also not significantly different between groups.

**Table-II:** Operation Characteristics and Perioperative Complications.

	Lymphadenectomy (Group A) (n=108)	No Lymphadenectomy (Group B) (n=72)	P value
Total number of complications	15 (13.9)	6 (8.3)	0.2
Wound infection	8 (7.4)	2 (2.7)	0.7
Thromboembolism	2 (1.8)	1 (1.3)	0.6
Other complications	5 (4.6)	3 (4.1)	0.4
Mean estimated blood loss (mL)	474	606	0.3
Median operating time (min)	160	141	0.05

Data are presented as number or number (percentage).

At a median follow-up of 26 months, 23 adverse events were observed. (Table-III). Endometrial cancer recurrence was observed in 10 patients (5.5% of all 180 patients). Of these, 9 patients were in Group A (9/108; 5.5%) and 1 was in the Group B (1/72; 1.3%). The most common sites of recurrence were the vaginal vault and lungs. Thirteen patients died during the study period: 7 patients (3.9%) in Group A and 6 patients (3.3%) in Group B.

**Table-III:** Adverse events.

	Lymphadenectomy (Group A) (n=108)	No Lymphadenectomy (Group B) (n=72)
Recurrence	9 (5)	1 (0.6)
Death	7 (3.9)	6 (3.3)

Data are presented as number (percentage).

**Fig.1:**
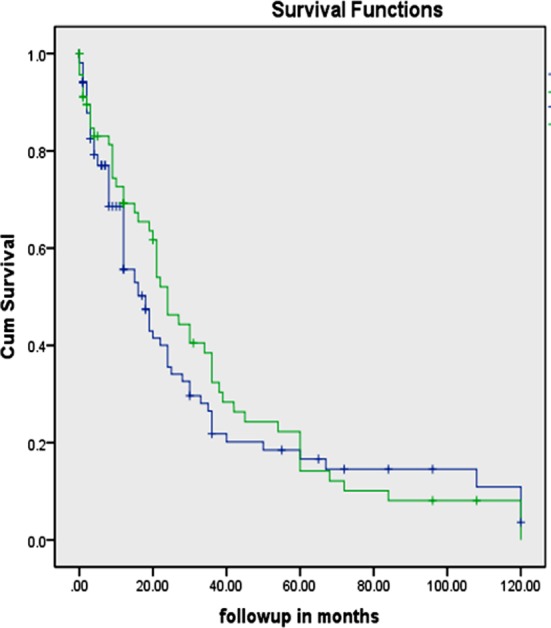
Overall survival comparison between the two study groups. Lymphadenectomy groupNo lymphadenectomy group.

Using the Kaplan-Meier method and log rank test, we found that patients in Group A had comparable overall survival to those in Group B (32 months versus 34 months; p = 0.47) (Fig.1). Using the same methods, overall survival was found to be 30 months for those with > 5 pelvic lymph nodes removed compared to 37 months for those with < 5 lymph nodes removed. This difference was not statistically significant (p = 0.70).

Survival was compared by univariate analysis in the two treatment arms to determine the effects of various covariates, including age, tumour grade, extent of myometrial invasion, performance of selective lymphadenectomy, and number of lymph nodes removed (Table-IV). The results showed that none of these factors were associated with survival.

**Table-IV:** Univariate analysis of overall survival for potential prognostic factors.

Prognostic factor	Overall Survival (months)	P-value
Lymphadenectomy	32	0.47
No lymphadenectomy	34
*Age group (years)*		
< 45	33.3	0.76
> 45	32.4
*Tumour grade*		
I	38	0.07
II	27
III	18.7
*Myometrial invasion*		
None	26.6	0.81
< 50%	32
> 50%	35.5
*Mean number of lymph nodes removed*		
<	37.3	0.70
5 > 5	29.8

## DISCUSSION

Endometrial cancer is one of the most common malignancies in females. The principal method of distant spread for this cancer is via the lymphatic route. The 1988 International Federation of Gynecology and Obstetrics (FIGO) staging system emphasizes performing complete surgical staging, including lymph node dissection, as the standard management for surgical staging of this malignancy. In the revised 2009 FIGO staging system, performing pelvic and para-aortic lymphadenectomy was noted to increase the prognostic value of staging even more than the 1988 system.^7^ According to the most recent National Comprehensive Cancer Network guidelines, pelvic node dissection is an important component for surgical staging, as it helps provide important prognostic information.^8^

There is a long-standing debate among gynaecologic oncologists regarding the benefits versus risks of selective lymphadenectomy, especially in early-stage endometrial carcinoma.^6^,^9^,^10^ This debate centres around the impact on survival, operating time, infection rates, costs, and risk of lymphoedema.^9^,^11^ The currently available literature does not provide definitive conclusions about the role of lymphadenectomy in early-stage endometrial carcinoma, therefore the issue remains controversial. This is reflected in the practice patterns of gynaecologic oncologists noted in a self-administered survey of members of the Society of Gynaecologic Oncology. Only 35% of the respondents evaluated both pelvic and para-aortic lymph nodes in early-stage disease.^12^ By contrast, a higher percentage (98%) of institutions in Japan routinely perform pelvic lymph node dissection in endometrial cancer.^13^

The risk of nodal involvement in early stage disease: In the current study, 5% of patients had pelvic lymph node metastases and only 1% had para-aortic involvement. These results are similar to the rates reported by Cragun et al. (5% pelvic and 3% para-aortic node involvement)^2^ and Zuurendonk et al. (5% positive lymph nodes).^14^ However, they are considerably lower than the 9% and 6% rates of positive pelvic and para-aortic lymph nodes, respectively, reported by the Gynecologic Oncology Group study of early-stage endometrial cancer patients,^15^ and the 13.3% rate of lymph node involvement reported in a randomized trial of systematic lymphadenectomy versus no lymphadenectomy.^3^

The node-positive patients were not equally distributed among the tumour grades in our study. The majority of node-positive patients had grade 2 or 3 histology. Endometrioid histology was observed in more than 75% of node-positive patients, implying that omission of lymphadenectomy in type 1 cancers is not advisable. Because selective lymphadenectomy upstaging to stage 3 and 4 was observed in 11% of patients in the lymphadenectomy group, performing lymphadenectomy affected the accuracy of the prognosis.

### Morbidity of the surgical procedure

Morbidities previously reported to be associated with pelvic and para-aortic lymphadenectomy include a significantly longer hospital stay, increased mean operative blood loss resulting in an increased rate of blood transfusions, lymphocysts, and lymphoedema.^9^,^11^,^16^ In our study, no significant surgical morbidity was associated with lymphadenectomy. Mean operative blood loss and duration of surgery were comparable between the lymphadenectomy and no lymphadenectomy groups.

### Survival impact of lymphadenectomy

Several authors have suggested a beneficial effect of lymphadenectomy on survival.^2^,^17^-^20^ Most of these were based on retrospective studies, in which selection bias and adjuvant treatment of patients with nodal metastases may have contributed to the improved survival.

More recently, the results of A Study in the Treatment of Endometrial Cancer (ASTEC), a randomized trial, have not supported a therapeutic benefit of lymphadenectomy.^4^ However, this trial has received much criticism because of its inclusion of tumours with non-endometrioid histology, lack of a centralized pathologic review process, and the use of postoperative radiation that was not based on lymph node status.^21^ Similar results were obtained by Dowdy et al., who reported no survival benefit of lymphadenectomy in a low-risk group based on the Mayo criteria (type 1 histology, grade 1 or 2, myometrial invasion < 50%, and primary tumour diameter ≤ 2 cm).^16^ Studies by Hidaka et al., Neubauer et al., and Wang et al. also suggested no overall survival benefit, but the latter two studies showed that lymphadenectomy was helpful in identifying patients with risk factors for tailoring adjuvant treatment.^9^,^22^,^23^ In the study by Wang et al.^23^, a small subset of patients with stage 1b showed improved survival with lymphadenectomy. In our study, no survival advantage was observed by performing lymphadenectomy in early-stage endometrial cancer.

### Role of intraoperative assessment regarding lymphadenectomy

Excellent disease-free survival, over 96%, has been reported by institutions in which lymph node dissections have been omitted from the management of low-risk endometrial cancers.^6^,^9^,^24^,^25^ Furthermore, three studies have been published in which omission of lymph node dissection was based on intraoperative assessment of tumour size, depth of myometrial invasion, and histologic grade.^16^,^25^,^26^ The intraoperative assessment was accomplished by gross examination and intraoperative frozen section. However, these institutions had documented reliable capabilities of frozen section assessment of pathologic specimens, which may not be available in all hospitals.

Effect of number of lymph nodes on survival: The optimum lymph node count associated with improved survival has also been a subject of considerable debate among researchers. Removal of 10 or more regional lymph nodes has been associated with improved survival in early-stage disease.^10^ This was one of the controversies regarding the ASTEC trial, as 9 or fewer lymph nodes were resected in 35% of the patients in that study. In the present study, the median number of pelvic lymph nodes removed was 9.

## CONCLUSION

Selective lymphadenectomy offers the advantage of better surgical staging, but it has no therapeutic benefit in terms of improving overall or disease-free survival. Perioperative morbidities are comparable, whether or not selective lymphadenectomy is performed.
